# A chromosome-level genome of *Semiothisa cinerearia* provides insights into its genome evolution and control

**DOI:** 10.1186/s12864-022-08949-z

**Published:** 2022-10-21

**Authors:** Shengqi Chi, Yanchun Wang, Zhongkai Wang, Haorong Li, Songdong Gu, Yandong Ren

**Affiliations:** 1 Key Laboratory of Integrated Crop Pest Management of Shandong Province, College of Plant Health and Medicine, Qingdao Agricultural University, Qingdao, 266109 China; 2 College of Science and Information, Qingdao Agricultural University, Qingdao, 266109 China; 3 School of Ecology and Environment, Northwestern Polytechnical University, Xi’an, 710072 China; 4 College of Life Sciences, Shaanxi Normal University, Xi’an, 710062 China

**Keywords:** *Semiothisa cinerearia*, Geometridae, *tipE*, Cytochrome P450

## Abstract

**Background:**

*Semiothisa cinerearia* belongs to Geometridae, which is one of the most species-rich families of lepidopteran insects. It is also one of the most economically significant pests of the Chinese scholar tree (*Sophora japonica* L.), which is an important urban greenbelt trees in China due to its high ornamental value. A genome assembly of *S. cinerearia* would facilitate study of the control and evolution of this species.

**Results:**

We present a reference genome for *S. cinerearia*; the size of the genome was ~ 580.89 Mb, and it contained 31 chromosomes. Approximately 43.52% of the sequences in the genome were repeat sequences, and 21,377 protein-coding genes were predicted. Some important gene families involved in the detoxification of pesticides (P450) have expanded in *S. cinerearia*. Cytochrome P450 gene family members play key roles in mediating relationships between plants and insects, and they are important in plant secondary metabolite detoxification and host-plant selection. Using comparative analysis methods, we find positively selected gene, *Sox15* and *TipE*, which may play important roles during the larval-pupal metamorphosis development of *S. cinerearia*.

**Conclusion:**

This assembly provides a new genomic resource that will aid future comparative genomic studies of Geometridae species and facilitate future evolutionary studies on the *S. cinerearia*.

**Supplementary Information:**

The online version contains supplementary material available at 10.1186/s12864-022-08949-z.

## Background

*Semiothisa cinerearia* belongs to one of the most species-rich families in Lepidoptera, the Geometridae, which contains approximately 21,500 species [1]. Geometridae comprises 9 subfamilies [2] and have a global distribution (with the exception of the polar regions). In addition, many of them are considered as pests. Among the major groups of genes help Geometridae adapting to various environments, Cytochrome P450 and Hsp (heat shock protein) genes are most important. These genes involved in plant secondary metabolite detoxification and tolerance to heat stress might be related to the invasiveness of some insects [3–5]. Studying the P450 and HSP gene repertoire in Geometridae will help us understand pests and control them [6–10]. One of most economically significant in Geometridae is *S. cinerearia*, which is the pests of important urban greenbelt trees Chinese scholar tree (*Sophora japonica* L.) [11]. Many researchers are working on the control of *S. cinerearia* in recent years [12, 13]. Pesticides are key for the control of it; however, the use of pesticides has had deleterious effects on urban ecosystems and its natural enemies. Although the ecology of *S. cinerearia* is well studied, genomic data are still lacking, and this has impeded further improvements of control.

Here, we used a hybrid sequencing approach combining Illumina short reads, Nanopore long reads, and Hi-C scaffolding to generate a chromosome-level genome assembly for *S. cinerearia*. Using Hi-C scaffolding, we assigned 97.64% of the contigs to 31 chromosomes. This genome assembly fills a major taxonomic gap in Geometridae comparative genomics and provides valuable clues for pest control. In this study, we explore the evolution of the cytochrome P450 and HSP gene families in related species, as well as several important genes that might be involved in pupal development in Geometridae species. Two positively selected genes, *Sox15* and *TipE*, may play important roles during the larval-pupal metamorphosis development of *S. cinerearia*. A complete genome will advance our understanding of the molecular mechanisms underlying processes of tolerance to insecticides and other abiotic stresses, and accelerate studies on *S. cinerearia* development, which will facilitate the control of *S. cinerearia* as a natural enemy.

## Results and discussion

### Genome sequencing and de novo assembly

**Fig. 1:**
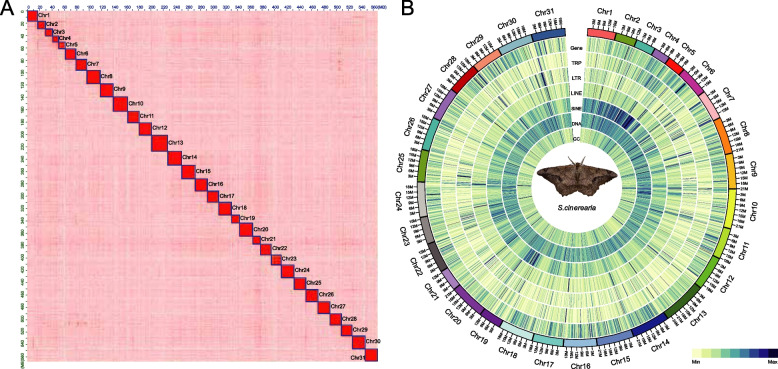
Chromosome-level assembly of *Semiothisa cinerearia. ***A** The genome‐wide Hi‐C interaction maps of 31 chromosomes in the *S. cinerearia* genome. Calculated interaction frequency distribution of Hi-C links between and within chromosomes. **B** Circos graph of characteristics of the *S. cinerearia* genome. From the outer ring to inner circle: marker distribution on 31 chromosomes at the megabase scale (I), gene distribution (II), tandem repeat (TRP) (III), long terminal repeat (LTR) (IV), long interspersed nuclear element (LINE) (V), short interspersed nuclear element (SINE) (VI), DNA elements (VII), and guanine-cytosine (GC) content (VIII)

A total of 48.34 Gb data of Illumina paired-end reads (coverage: 83.22 ×) were obtained for the genome survey, genome assembly, and other related analyses (Table S1). The size of the *S. cinerearia* genome estimated using 17-mer analyses was ~ 667.93 Mb (Figure S1). The primary genome assembly for *S. cinerearia* was performed using the 40.45 Gb Oxford Nanopore long reads (coverage: 69.64 × , Table S2) and further polished using Illumina paired-end reads. A draft genome assembly of 580.85 Mb was obtained, which yielded 450 contigs with a contig N50 of 4.15 Mb (Table S3). A total of 50.54 Gb Hi-C reads (coverage: 87.01 ×) were used to orient and anchor 450 contigs to 31 chromosomes (Fig. 1A, Tables S3 and S4). The Hi-C linking information indicated that more than 97.64% of the assembled bases were anchored to the chromosomes (Table S5). The N50 results suggest that the assembly is highly contiguous.

For evaluating assembly quality, all RNA-seq reads were assembled into transcripts (Tables S6 and S7). A total of 98.40% of the assembled transcripts (58,742 of 59,695) could be mapped to the assembled genome (Table S8), indicating its high completeness. A BUSCO assessment showed that 94.70% of Lepidoptera core genes were successfully detected in the assembled genome (Table S9). Synteny analyses between *S. cinerearia* and *Operophtera brumata* revealed only 2 chromosomes (chromosome 12 and 14 in *S. cinerearia*) showing strong synteny, most of the chromosomes have no clear patterns of orthology (Figure S2), highlighting a high degree of genome structure evolution in Geometridae. The results of these approaches indicated that the genome assembly was complete and suitable for subsequent analysis. The assembled genome was also compared with the genomes of other related species (Table S10). This genome is the second chromosome-level genome assembly in Geometridae. Compared with other related species, the N50 of *S. cinerearia* (19.57 Mb) is in the same order of magnitude to that of other chromosome-level Lepidopteran assemblies (65.63 Kb-27.09 Mb). Additionally, the BUSCO complement of *S. cinerearia* (96.5%), shows comparable gene-completeness to that of other high quality Lepidopteran assemblies (from 78.8% to 99.2%) (Table S10). These results showed that the assembled *S. cinerearia* genome had a high level of continuity and completeness.

### Genome annotation

**Fig. 2:**
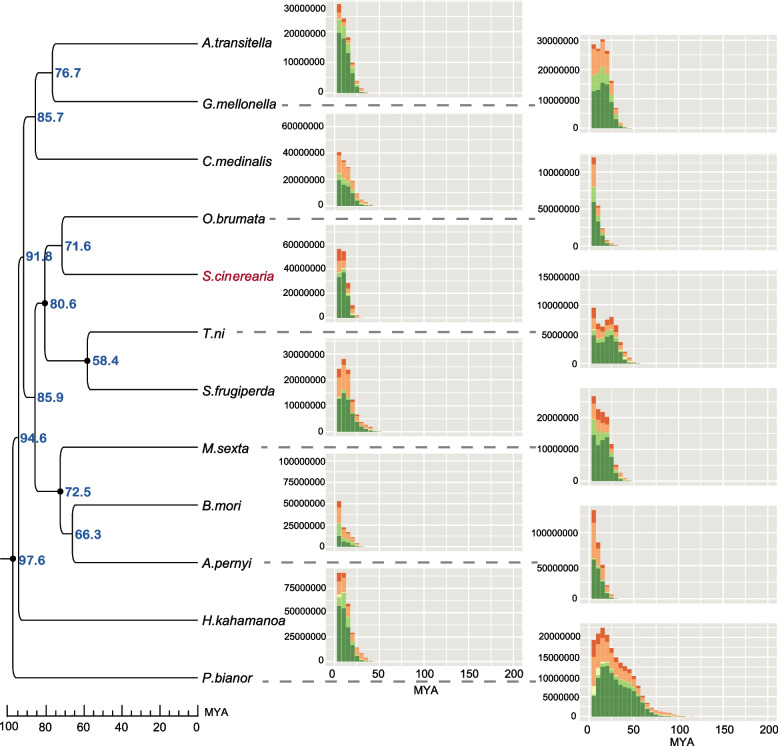
Comparison of TEs insertion history among species. The x-axis indicates the inferred insertion time (unit: million years ago) of TEs. The y-axis indicates the length of different TEs elements

A combined structure- and homology-based analysis identified a total of 252.83 Mb repetitive sequences, accounting for 43.52% of the total *S. cinerearia* genome (Table S11). Among these repeats, DNA elements are the most abundant (7.62%), followed by long terminal repeats (LTRs) (6.46%), Short interspersed nuclear elements (SINEs) (2.46%), and long interspersed nuclear elements (LINEs) (2.26%) (Table S12). The GC content, gene density, and the distribution of all TEs of *S. cinerearia* are shown in Fig. 1B. We also assessed the types of TEs and the TEs insertion times of the 12 studied species (*S. cinerearia, O. brumata*, *Papilio bianor*, *Cnaphalocrocis medinalis*, *Hyposmocoma kahamanoa*, *Spodoptera frugiperda*, *Galleria mellonella*, *Amyelois transitella*, *Bombyx mori*, *Manduca sexta*, *Trichoplusia ni*, and *Antheraea pernyi*) and found that the TEs insertion events in Geometridae (*S. cinerearia* and *O. brumata*) occurred 5–10 Mya. The TEs insertion times of all Geometridae are much more recent than the divergence between them, which suggests that TEs insertion events might have occurred after their divergence (Fig. 2). The DNA elements proportion was much higher in *S. cinerearia* (19.76%) compared with that in other insects (from 5.51% to 17.24%) (Figure S3). However, the DNA elements proportion of another insect in Geometridae (*O. brumata*) was only 7.16%, which indicates that the DNA elements proportion in different species of Geometridae are quite different. In addition, the other TEs types in *S. cinerearia* and *O. brumata* are also different. The proportion of LINE, LTR, and SINE elements in *S. cinerearia* are 16.75%, 5.85%, and 6.38%. However, the proportion of LINE, LTR, and SINE elements in *O. brumata* are 25.39%, 0.92%, and 17.39%. These differences in the proportion of TEs types in the family suggest that changes are not conserved in Geometridae and might be specific to the *S. cinerearia* lineage.

To annotate the genes in the draft *S. cinerearia* genome, we integrated ab initio, homology‐, and transcript‐based gene identification approaches. A total of 21,377 protein-coding genes were detected in the *S. cinerearia* genome. The mRNA length, CDS length, exon length, and exon number distribution of *S. cinerearia* are similar to other Lepidopteran species (Figure S4). The quality of the protein-coding gene annotations was comparable to that in previous studies of other species. Among all predicted genes, functional annotations based on the NCBI Nr databases [14], TrEMBL [15], InterProScan [16], GO (gene ontology: http://geneontology.org/), COG (https://www.ncbi.nlm.nih.gov/COG/), Swiss-Prot (www.uniprot.org), and Kyoto Encyclopedia of Genes and Genomes [17–19] could be assigned to 86.41% (18,472) of the 21,377 genes (Table S13). The functional annotation results implied that most of the protein-coding genes in *S. cinerearia* can find homolog genes in public database. Overall, these findings indicate the high accuracy and completeness of the predicted gene models.

### Genome evolution

**Fig. 3:**
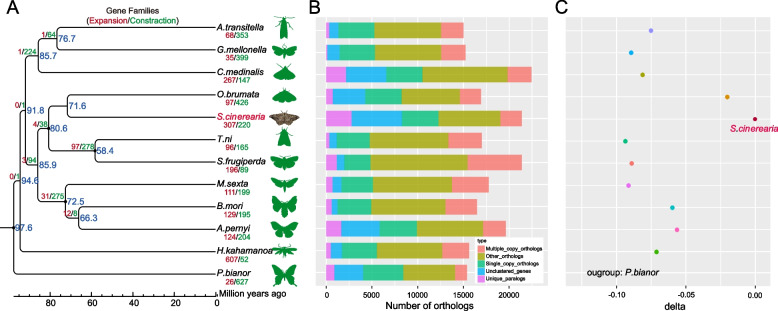
Orthology and evolutionary analysis among species. **A** Species phylogenetic tree and gene family expansion/contraction. The phylogenetic relationships of *S. cinerearia* with other insects were characterized using a maximum likelihood analysis of a concatenation of single‐copy orthologous protein sequences and 100 bootstrap replicates. The numbers of gene families that underwent expansion (red) or contraction (green) are shown on branches with predicted species divergence times plotted at each node. **B** Orthologous genes distribution. The bars are subdivided to indicate different orthologous relationships. Single-copy and multiple-copy orthologs are families in which each species has only one or more than one copy, respectively. Other orthologs are genes with matches with other species that could not be placed into other ortholog categories. Unclustered genes are genes in each species that could not be associated with the gene predictions in any of the other lineages. Unique paralogs show species-specific families. **C** Relative evolutionary rates of these species. The analysis was performed using single-copy protein-coding genes with *S. cinerearia* as the reference species and *P. bianor* as the outgroup species. The x-axis indicates the relative evolutionary rates of the species

To evaluate the evolutionary conservation of the *S. cinerearia* genome relative to other insect species, we compared reported gene sets of 12 insect species. All the 1,149 single-copy orthologs were aligned to build a super-sequence and construct a phylogenetic tree. Phylogenetic inference confirmed that *S. cinerearia* and *O. brumata* were sister group to Noctuidae, which supported the grouping of Glossata. The Geometridae lineage was estimated to diverge from the Noctuidae lineage ~ 80.6 Mya (confidence intervals: 63.0–97.5 Mya) according to MCMCTree (Fig. 3A; Figure S5). A total of 17,884 gene family clusters were constructed, of which 2,772 belong to unique gene families (Fig. 3B).

Based on the previous results, the gene family expand analysis was performed by CAFÉ (v4.1) using default parameters. Gene family expansion often associated with the adaptive divergence of closely related species. To investigate the key genomic changes in *S. cinerearia* associated with adaptation, expanded gene families in *S. cinerearia* were identified. We detected 307 gene families that have undergone expansions, and GO enrichment analysis of these expanded gene families suggests that most of these genes are involved in metabolic process and biosynthetic process (Fig. 3A, Table S14). The results of KEGG enrichment analysis were consistent with these findings (Table S15). Interestingly, both GO and KEGG enrichment analysis indicated these genes involved in biosynthesis of cutin (map00073, *p*-value = 0.000441887) and structural constituent of cuticle (GO: 0,042,302, *P*-value = 1.18E-08) have expanded in *S. cinerearia*. These results suggests the cutin biosynthesis pathways in *S. cinerearia* is different from other inserts and more genes are involved in biosynthesis of cutin. It is generally known that the cuticle is the exoskeleton of insect, and also considered as an adaptable tissue that can determine the physiological development and defend against different environmental stresses, especially for dehydration, parasites, and pesticides [20, 21]. As the outer waterproofing materials, cuticular hydrocarbons and lipids are involved in contributing to the penetration insecticide resistance [22]. The insect cuticle composed primarily of chitin, proteins, catecholamines, lipids and its biosynthesis pathways is quite complex. The cutin biosynthesis genes in *S. cinerearia* were not reported before, however, the gene number variation in different insect were reported [20, 23–26]. The cutin biosynthesis genes in different insects may help them adapt to different environments stresses, the expanded cutin biosynthesis genes may also help *S. cinerearia* adapt to its environments stresses.

### Rate of molecular evolution

We found that the branch length of *S. cinerearia* was longer than that of other insect species (Figure S6), suggesting that the rate of protein evolution is higher in *S. cinerearia* compared with other insect species analyzed in this study. Relative rate tests and two cluster analysis confirmed that the rate of protein evolution of *S. cinerearia* was significantly higher compared with that of the other insect species (Tables S16 and S17). The fastest evolutionary rate was observed in Geometridae (*S. cinerearia* and *O. brumata*); however, Noctuidae (*S. frugiperda* and *T. ni*) and Sphingidae (*M. sexta*) have the slowest evolution rate (Fig. 3C). The molecular mechanisms of the fast molecular evolutionary rate in Geometridae remain unclear. Variation in the rate of molecular evolution is manifest at different species due to variation mutation rate and substitution rate [27]. These two reasons may cause the fast molecular evolutionary rate of Geometridae species and more genome data are needed for further check.

### Positively selected genes in Geometridae

**Fig. 4:**
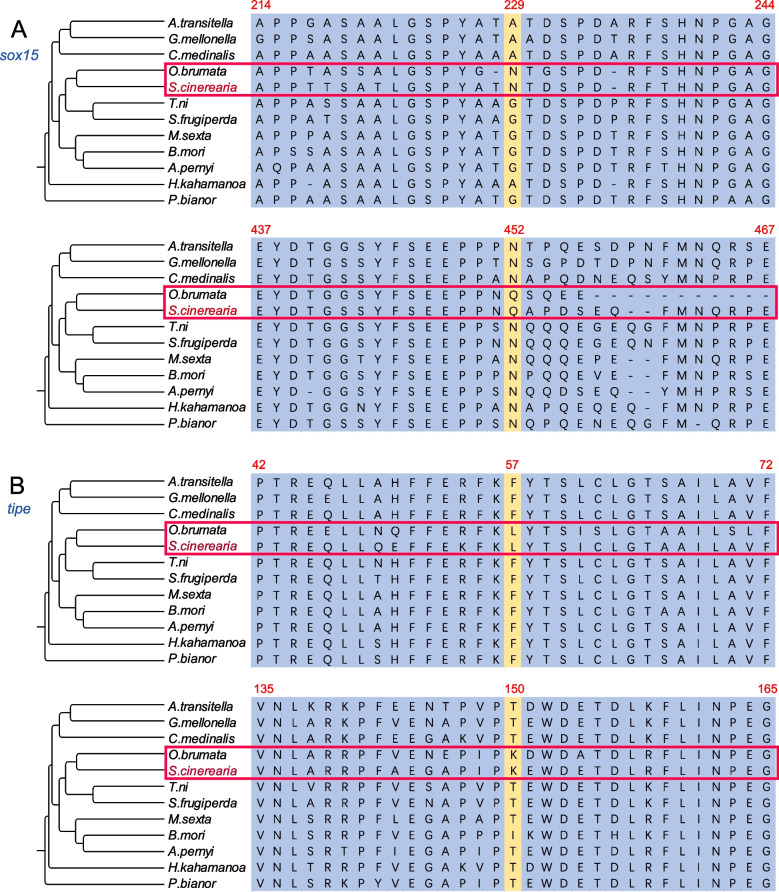
Positively selected sites of *sox15* and *tipE* in various insect species.** A** Positively selected sites in *sox15* gene. The left panel is the phylogenetic tree of these species, and the right panel is the amino acids sequences of *sox15* gene. **B** Positively selected sites in *tipE* gene. The left panel is the phylogenetic tree of these species, and the right panel is the amino acids sequences of *tipE* gene

We identified 34 positively selected genes (PSGs) in the Geometridae lineage, and these genes are most related to some biosynthesis and metabolic pathway (Table S18). Interestingly, *TM-A2B* (Larval cuticle protein A2B), a component of the cuticle, was undergoing positive selection. This result is consistent with expanded genes in *S. cinerearia*, the genes involved in biosynthesis of cutin not only expanded but also under positively selection in Geometridae. Chitinous structures are physiologically fundamental in insects, and are target sites for the development of new insect-pest-control strategies [28–30]. Further studying on this gene may help us develop new means of pest control. We also identified the two PSGs *Sox15* (*SoxF*) and *TipE*, which each had two positively selected sites (position 229 and 452, Fig. 4A; position 57 and 150, Fig. 4B, respectively). The *Sox15* gene has been shown to be involved in wing disc development in *Drosophila melanogaster* [31]. This gene might play an important role in wing disc development of larval-pupal metamorphosis. For *TipE* gene, the previous studies proved that *TipE* is functionally related to sodium channels [32], and sodium channels can affect spike shape and dendrite growth during postembryonic maturation [33]. Therefore, *TipE* gene might play a key role in pupal development of Geometridae species. *S. cinerearia* is a holometabolous insect, and these two genes may related to the larval-pupal metamorphosis development. More importantly, many studies showed that the larval-pupal metamorphosis is a very complex process, some key genes involved in larval-pupal metamorphosis could be used as targets for pest management [34–37]. These genes may become an important breakthrough for pest control of *S. cinerearia*.

### Cytochrome P450

**Fig. 5:**
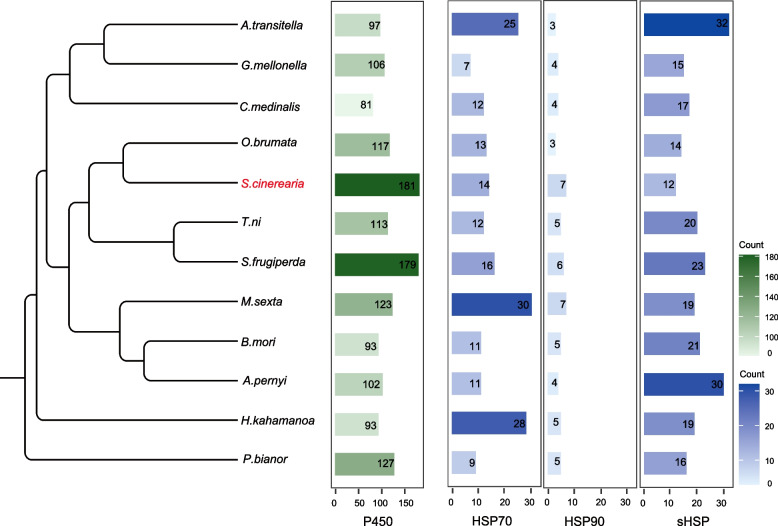
Number of cytochrome P450 gene, HSP70, HSP90, and sHSP genes in various insect species. The left panel is the phylogenetic tree of these species, the right panel is the gene number of P450, HSP70, HSP90, and sHSP genes

To facilitate studies of insecticide resistance, cytochrome P450 monooxygenase (P450s), which are major detoxification enzymes, were annotated in all these 12 species. The cytochrome P450 gene family is involved in host-plant adaptation. P450 enzymes, which are a large family in insect genomes, are involved in the detoxification of plant toxins and play a key role in insecticide resistance [38]. The number of P450 genes in *S. cinerearia* is 181, which is higher compared with the number of P450 genes in other studied Lepidopterans (81–179). The number of P450 genes of *S. cinerearia* (181) is higher than that in other Lepidopterans species, the number of P450 genes in *O. brumata* (117) is closed to other Lepidopterans species (Fig. 5), which showed the representative for a specific detoxification gene repertoire in *S. cinerearia*. The study of *O. brumata* genome data also showed the similar results [39].

### Heat shock proteins

Hsps represent a supergene family and can be usually divided into several families, including Hsp90, Hsp70, Hsp60, Hsp40 and small heat shock proteins (sHsps, molecular weights ranging from 12 to 43 kDa) [40, 41]. They are important molecular chaperones that are involved in thermal adaptation and resistance to some proteotoxic stresses [42]. More importantly, the altered expression of Hsps was accompanied with larva-to-adult survival reduction [43]. The number of HSP70 gene in Geometridae are 13 (*O. brumata*) and 14 (*S. cinerearia*), which is close to other species (from 7 to 30). The gene number of HSP90 in *O. brumata* and *S. cinerearia* are 3 and 7, respectively. The gene number of sHSP in *O. brumata* and *S. cinerearia* are 14 and 12, respectively. Overall, the number of HSP genes was not higher in Geometridae compared with that in other insects (Fig. 5), suggesting that HSP genes have not expanded. The lack of expansion of HSP genes indicating that the P450 genes, not the HSP genes, may helpful for the invasiveness and development of Geometridae. These results are consistent with the previous study that P450 gene family expanded in another Geometridae species, *O. brumata* [39].

## Conclusions

In this study, we sequenced and assembled the chromosome-level genome of *S. cinerearia* using Illumina sequencing, Nanopore sequencing, and Hi-C technology. The high-quality genome assembly of *S. cinerearia* will facilitate future genome-level investigations of various aspects of the biology of this pest as well as comparative evolutionary studies of *S. cinerearia* and other Geometridae insects. This important genomic resource data thus makes a major contribution to invasion biology research and the study of geometrid moths.

The size of the *S. cinerearia* assembly was approximately 580.89 Mb; it contained 31 chromosomes, and the N50 was ~ 19.57 Mb. Various analyses indicated that the completeness of the genome assembly was high and thus that it was suitable for subsequent analysis. Based on this assembly, 252,828,304 bp repeat sequences (43.52%) and 21,377 protein-coding genes were identified. Divergence time analysis showed that *S. cinerearia* diverged from the common ancestor with *O. brumata* at 71.6 Mya (confidence intervals: 54.7–88.1 Mya). The structural constituents of cuticle gene families have undergone a significant expansion in the *S. cinerearia* genome. In addition, some important gene families involved in the detoxification of pesticides (P450) have expanded in *S. cinerearia*. Using comparative analysis methods, we found that two positively selected genes *sox15* and *TipE*. *Sox15* gene might play an important roles in wing disc development of larval-pupal metamorphosis development stage. *TipE* gene might play a key role in pupal development of Geometridae species. Due to the essential larval-pupal metamorphosis development stage in *S. cinerearia*, these 2 genes are important for its development and are critical for its control. We studied and identified the important genes in *S. cinerearia* and argue its link for its development, providing a reference for future studies in *S. cinerearia* for its control.

## Methods

### Sampling and sequencing

The pupae of *S. cinerearia* was collected in Haiyang County, Yantai City, Shandong Province, China, in June 2021 from soil under a Chinese scholar tree (*Sophora japonica* L.). To avoid genome contamination from other organisms, such as microbes, *S. cinerearia* was rinsed with distilled water for 2 min. Genome DNA extraction was performed using a Blood & Cell Culture DNA mini kit (Qiagen, Germany). Total RNA was extracted using a TRIzol kit (Life Technologies, USA). Extracted DNA that met quality and quantity standards was split into three aliquots, which were used to construct an Oxford Nanopore (PromethION) library, an Illumina NovaSeq 6000 library, and a Hi-C library (Illumina NovaSeq 6000 platform). The RNA sequencing library was constructed and sequenced on the Illumina NovaSeq 6000 platform.

### Genome assembly and validation

For Illumina paired-end sequenced raw reads, including the genomic short-insert reads, Hi-C sequencing reads, and RNA-seq reads, adaptors and low-quality reads were removed by FASTX_Toolkit (http://hannonlab.cshl.edu/fastx_toolkit/commandline.html#fastq_quality_filter_usage). For Nanopore reads, all reads with an average quality ≥ 7 were retained for genome raw assembly using a Perl script (https://ftp.cngb.org/pub/gigadb/pub/10.5524/102001_103000/102210/filter_ONT_data_for_7_with_auto_check_quality_position_wzk.pl). The genome size of the *S. cinerearia* genome was estimated by filtered Illumina reads, using *17-mer* analysis. The genome size was estimated using the total number of 17-mers divided by G = K-num/K-depth (where K-num is the total number of 17-mers, K-depth denotes the k-mer depth and G represents the genome size) [44].

The filtered Nanopore long reads were used for de novo genome assembly with NextDenovo (V2.4, https://github.com/Nextomics/NextDenovo) with default parameters. To improve genome quality, the Illumina short-insert reads were used to polish the genome using NextPolish (v1.4.0) with default parameters. All Hi-C reads were used for chromosome construction. 3D DNA (v180419, https://github.com/aidenlab/3d-dna) was used to cluster, order, and orient the contigs into pseudo-chromosome sequences.

The quality of the reference genome sequence was also evaluated using BUSCO software (V5.2.2) [45] with the core gene set of the eukaryote, metazoan and lepidoptera databases, respectively. Illumina reads were mapped to the genome using BWA software (v0.7.12) [46] and the assembled transcripts were mapped to the genome using BLAT software [47]. Genome synteny between *S. cinerearia* and *O. brumata* was investigated using LAST software (version 802) with default parameters [48] and plotted using Circos (v0.69–6) software [49].

### Repetitive elements annotation

A de novo repeat database was first built using RepeatModeler (v-1.0.11) (http://www.repeatmasker.org/RepeatModeler/), and RepeatMasker (v-4.1.0) was applied to produce a homolog-based repeat library with default parameters. After combining the de novo repeat database and homolog-based repeats in Repbase, comprehensive repeat and TE detection was conducted using RepeatMasker (v1.323) [50] and RepeatProteinMask (v1.36). Tandem repeats in the genome were analyzed using Tandem Repeat Finder (v4.09) (v4.09) [51] with default parameters. The divergence level (Kimura) of a repeat copy to its consensus sequence was estimated using RepeatMasker, and the insertion time (T) of repeats was estimated using the formula T = k/2r using custom Perl scripts (https://github.com/4ureliek/Parsing-RepeatMasker-Outputs) [52].

### Protein-coding gene annotation

Protein-coding genes of *S. cinerearia* were predicted using ab initio prediction, as well as homology‐ and transcript‐based approaches. For ab initio prediction, Augustus (v3.3), SNAP (release: 2006–07-28), and Genescan were used for ab initio prediction. For homology-based prediction, the protein sequences of *Operophtera brumata* (https://www.bioinformatics.nl/wintermoth/portal/data), *Papilio bianor* (http://gigadb.org/dataset/view/id/100653/File_page), *Cnaphalocrocis medinalis* (http://www.insect-genome.com/Cmed/), *Hyposmocoma kahamanoa* (GCA_003589595.1), *Spodoptera frugiperda* (GCF_011064685.1), *Galleria mellonella* (GCF_003640425.2), *Amyelois transitella* (GCF_001186105.1), *Bombyx mori* (GCF_014905235.1), *Manduca sexta* (GCF_014839805.1), *Trichoplusia ni* (GCF_003590095.1), and *Antheraea pernyi* (https://ngdc.cncb.ac.cn/search/?dbId=gwh&q=Antheraea+pernyi) were downloaded from NCBI and other related public databases. Protein sequences were searched in the *S. cinerearia* genome using blastp in BLAST (v2.2.28) [53], and then identified using GeneWise (v2.2.0) [54]. For transcript-based annotation, the assembled transcripts were mapped to the genome for gene structure prediction using PASA (v2.1). Lastly, EVM (v1.1.1) was used to integrate the predicted genes and generate a consensus gene set.

To assign functions to the newly annotated genes in the *S. cinerearia* genome, all gene sequences were searched using BLAST (v2.2.28) [53] with an e-value threshold of 1e^−5^ against the SwissProt, NCBI nonredundant amino acid sequences (NR), Gene Ontology (GO) [55], Translated EMBL-Bank (Trembl), and Kyoto Encyclopedia of Genes and Genomes (KEGG) databases [17–19]. We also annotated motifs and domains using Interproscan (version 5.27) [56] with publicly available databases, including Gene3D, PRINTS, Pfam, CDD, SMART, MobiDBLite, and PROSITE.

### Phylogenetic tree construction and divergence time estimation

Protein sequence data sets from 12 species (*H. kahamanoa* (GCA_003589595.1), *C. medinalis* (http://www.insect-genome.com/Cmed/), *A. transitella*, *B. mori*, *P. bianor*, *O. brumata*, *A. pernyi*, *T. ni*, *G. mellonella*, *S. frugiperda*, *S. cinerearia*, and *M. sexta*) were downloaded from NCBI and other databases. Redundant alternative splicing events were filtered to generate a single transcript for each protein set and aligned pair-wise to identify conserved orthologues using Blastp (E-value ≤ 1 × 10 − 5). The Blastp results were used in Orthmcl (v2.0.9) [57] to cluster gene families; orthologous single-copy genes were aligned using MUSCLE (v3.8.31) [58] with default parameters. Phylogenetic reconstruction of the 12 species was performed with RAxML (v8.2.10) [59] using this super-sequence and *P. bianor* as an outgroup species. Species divergence times were estimated using the MCMCTree program in PAML (v4.9) [60] based on 4d sites extracted using in-house scripts. The fossil records of these species were downloaded from TIMETREE website (http://www.timetree.org) for calibration in this step.

### Analysis of gene family expansion and contraction

Following gene family clustering and divergence estimation, the expansion and contraction of gene families were analyzed using CAFÉ (v4.1) with a probabilistic graphical model (PGM) to calculate the probability of transition in each gene family from parent to child nodes in the phylogeny. The expanded genes families in *S. cinerearia* were subjected to GO and KEGG enrichment analysis. GO enrichment analysis was conducted using the EnrichGO package in R (3.2.5). An R script was used for KEGG enrichment analysis [61, 62].

### Analysis on molecular evolution rate

The aligned protein sequences of the single-copy genes were used to calculate relative evolutionary rates via two methods: Tajima’s relative rate test and two cluster analysis. MEGA software (v10) [63] was used for Tajima’s relative rate test. *P. bianor* was used as the outgroup species, and the relative evolutionary rates between *S. cinerearia* and other species were calculated. A Chi-square test was used to identify species with faster evolutionary rates. Two cluster analysis was conducted in LINTRE software [64] using the TPCV model. We also specified *P. bianor* as the outgroup species and determined the relative evolutionary rates between *S. cinerearia* and other species.

### Positive selection analysis

Single-copy genes identified by Orthomcl software among the 12 species were extracted and aligned using MUSCLE software (v3.8.31) [58] with default parameters to identify potential positively selected genes (PSGs), using branch model in the Codeml tool of the PAML package (v4.8) [65]. At first, the rate ratio (ω) of nonsynonymous to synonymous nucleotide substitutions was estimated. The one-ratio model was used to detect average ω across the species tree (ω0). For each gene, the two-ratio branch model was used to detect the ω of the appointed branch (Geometridae) to test the (ω1) and ω of all other branches (ω_background). A likelihood ratio test was performed to compare the fit of the two-ratio models with the one ratio model to determine whether the gene was positively selected in the appointed branch (ω1 > ω0; ω1 > ω_background; ω1 > 1; *P*-value < 0.05).

## Supplementary Information

**Addition file 1: Table S1.** The statistics of sequencing reads on Illumina platform. These data are produced by short insert library, and the results were shown by the raw sequencing reads. The sequencing depth was calculated by the assembled genome size, the genome is 580,885,107 bp. **Table S2.** The statistics of sequencing reads on Nanopore platform. The reads with quality value Q > 7 were considered. The sequencing depth was calculated by the assembled genome size, the genome is 580,885,107 bp. **Table S3.** The statistics of the contig-level genome and chromosome-level genome. These data are produced by short insert library, and the results were shown by the raw sequencing reads. The contig-level genome was assembled by Nexedenovo and polished by Nextpolish. The chromosome-level genome is constructed by *3D* DNA. **Table S4.** The statistics of Hi-C sequencing reads. The sequencing depth was calculated by the assembled genome size, the genome is 580,885,107 bp. **Table S5.** Statistics of the assembled chromosome-level genome via 3D *de novo *assembly software. **Table S6.** The statistics of RNA sequencing reads on Illumina platform. These data are produced by short insert library, and the results were shown by the filtered reads. **Table S7.** The statistics of the assembled transcripts by Bridger of 5 organs/tissues. **Table S8.**The statistics of the transcripts mapping ratio on the assembled genome. **Table S9. **The quality evaluation of assembled genome by BUSCO software. **Table S10.** Comparison of related species genomes with our chromosome-level genome. **Table S11.** The statistics of the annotated repeat sequences in our assembled genome. The type represents that the way or software used in this study. **Table 12.** The statistics of the annotated repeat sequences in our assembled genome by *de novo* prediction. **Table S13.** The functional annotation of the predicted protein-coding genes. **Table S14.** GO enrichment of the expanded gene families in *S. cinerearia* analyzed by CAFÉ (v4.1). **Table S15.** KEGG enrichment of the expanded gene families in *S. cinerearia *analyzed by CAFÉ (v4.1). **Table S16.** Relative evolution rate among these species by LINTRE software. **Table S17.** Relative evolution rate among these species by MEGA software. **Table S18.** Statistics of positively selected genes in Geometridae. **Figure S1.** 17-mer analysis of *S. cinerearia* genome. **Figure S2.** Whole genome synteny analyses between *S. cinerearia* and *O. brumata*. **Figure S3.** TEs ratio in these species. **Figure S4.** Distribution of gene parameters in these species. **Figure S5.** Divergence time of these species. **Figure S6.** Phylogenetic relationship among the 12 species inferred by the nucleotide acid sequences of the single-copy genes. Number in the node represents the corresponding bootstrap value.

## Data Availability

The raw genome, transcriptome and Hi-C data in this study have been deposited with links to BioProject accession number PRJNA830997 in the NCBI BioProject database (https://www.ncbi.nlm.nih.gov/bioproject/PRJNA830997). The assembled genome was deposited in the Genome Warehouse in National Genomics Data Center, under accession number GWHBJXE00000000 that is publicly accessible at https://ngdc.cncb.ac.cn/gwh/Assembly/26019/show.

## Abbreviations

BUSCO: Benchmarking Universal Single-Copy Orthologs
TEs: Transposable elements
GO: Gene Ontology
KEGG: Kyoto Encyclopedia of Genes and Genomes
Mya: Million years ago
NR: Non-redundant protein
BWA: Burrows-Wheeler aligner
PSGs: Positively selected genes
