# Supplementary material for: A chromosome-level genome of Semiothisa cinerearia provides insights into its genome evolution and control
Source: BMC Genomics. 2022 Oct 21;23:718. doi: 10.1186/s12864-022-08949-z (PMC9585740; doi:10.1186/s12864-022-08949-z)
Supplement: Supplementary file 1 — Addition file 1: Table S1. The statistics of sequencing reads on Illumina platform. These data are produced by short insert library, and the results were shown by the raw sequencing reads. The sequencing depth was calculated by the assembled genome size, the genome is 580,885,107 bp. Table S2. The statistics of sequencing reads on Nanopore platform. The reads with quality value Q > 7 were considered. The sequencing depth was calculated by the assembled genome size, the genome is 580,885,107 bp. Table S3. The statistics of the contig-level genome and chromosome-level genome. These data are produced by short insert library, and the results were shown by the raw sequencing reads. The contig-level genome was assembled by Nexedenovo and polished by Nextpolish. The chromosome-level genome is constructed by 3D DNA. Table S4. The statistics of Hi-C sequencing reads. The sequencing depth was calculated by the assembled genome size, the genome is 580,885,107 bp. Table S5. Statistics of the assembled chromosome-level genome via 3D de novo assembly software. Table S6. The statistics of RNA sequencing reads on Illumina platform. These data are produced by short insert library, and the results were shown by the filtered reads. Table S7. The statistics of the assembled transcripts by Bridger of 5 organs/tissues. Table S8.The statistics of the transcripts mapping ratio on the assembled genome. Table S9. The quality evaluation of assembled genome by BUSCO software. Table S10. Comparison of related species genomes with our chromosome-level genome. Table S11. The statistics of the annotated repeat sequences in our assembled genome. The type represents that the way or software used in this study. Table 12. The statistics of the annotated repeat sequences in our assembled genome by de novo prediction. Table S13. The functional annotation of the predicted protein-coding genes. Table S14. GO enrichment of the expanded gene families in S. cinerearia analyzed by CAFÉ (v4.1). Table S15. KEGG [file 12864_2022_8949_MOESM1_ESM.docx]

**Table S1. The statistics of sequencing reads on Illumina platform.** These data are produced by short insert library, and the results were shown by the raw sequencing reads. The sequencing depth was calculated by the assembled genome size, the genome is 580,885,107 bp.

| Term | Read number | Total bases | Sequencing strategy | Sequencing depth (×) |
| --- | --- | --- | --- | --- |
| AM593-01R0001 | 322,277,372 | 48,341,605,800 | PE150 | 83.22 |
| Total | 322,277,372 | 48,341,605,800 | - | 83.22 |

**Table S2. The statistics of sequencing reads on Nanopore platform.** The reads with quality value Q > 7 were considered. The sequencing depth was calculated by the assembled genome size, the genome is 580,885,107 bp.

| Lib-ID | Mean length | N50 length | Read number | Total base | Sequencing depth (×) |
| --- | --- | --- | --- | --- | --- |
| ONT_BMK210827-AN332 | 21,697 | 26,944 | 1,864,320 | 40,450,492,659 | 69.64 |
| Total | 21,697 | 26,944 | 1,864,320 | 40,450,492,659 | 69.64 |

**Table S3. The statistics of the contig-level genome and chromosome-level genome.** These data are produced by short insert library, and the results were shown by the raw sequencing reads. The contig-level genome was assembled by Nexedenovo and polished by Nextpolish. The chromosome-level genome is constructed by *3D* DNA.

|  | **Contig-level genome** | | **Chromosome-level genome** | |
| --- | --- | --- | --- | --- |
| Term | Size (bp) | Number | Size (bp) | Number |
| N90 | 556,899 | 175 | 12,484,278 | 28 |
| N80 | 1,312,744 | 110 | 16,792,882 | 24 |
| N70 | 2,278,700 | 77 | 17,753,627 | 20 |
| N60 | 3,152,668 | 55 | 18,540,835 | 17 |
| N50 | 4,151,657 | 39 | 19,566,149 | 14 |
| Max length (bp) | 17,156,115 | - | 26,128,831 | - |
| Total size (bp) | 580,846,607 | - | 580,885,107 | - |
| Total number (>100 bp) | - | 450 | - | 105 |
| Total number (>10 kb) | - | 450 | - | 101 |

**Table S4. The statistics of Hi-C sequencing reads.** The sequencing depth was calculated by the assembled genome size, the genome is 580,885,107 bp.

| Term | Read number | Total bases | Sequencing strategy | Sequencing depth (×) |
| --- | --- | --- | --- | --- |
| BMK210827-AN332-ZX01-0201 | 336,948,526 | 50,542,278,900 | PE150 | 87.01 |
| Total | 336,948,526 | 50,542,278,900 | - | 87.01 |

**Table S5.** Statistics of the assembled chromosome-level genome via 3D *de novo* assembly software.

| Chromosome ID | Length (bp) | Percentage (%) | Chromosome ID | Length (bp) | Percentage (%) |
| --- | --- | --- | --- | --- | --- |
| Chr1 | 16,792,882 | 2.89% | Chr_17 | 18,363,252 | 3.16% |
| Chr_2 | 12,484,278 | 2.15% | Chr_18 | 20,570,461 | 3.54% |
| Chr_3 | 11,568,756 | 1.99% | Chr_19 | 13,197,689 | 2.27% |
| Chr_4 | 9,747,330 | 1.68% | Chr_20 | 21,084,642 | 3.63% |
| Chr_5 | 10,703,027 | 1.84% | Chr_21 | 12,630,832 | 2.17% |
| Chr_6 | 17,391,209 | 2.99% | Chr_22 | 17,753,627 | 3.06% |
| Chr_7 | 17,627,428 | 3.03% | Chr_23 | 15,987,569 | 2.75% |
| Chr_8 | 21,803,457 | 3.75% | Chr_24 | 20,789,992 | 3.58% |
| Chr_9 | 21,033,697 | 3.62% | Chr_25 | 19,035,630 | 3.28% |
| Chr_10 | 23,508,736 | 4.05% | Chr_26 | 19,566,149 | 3.37% |
| Chr_11 | 18,419,586 | 3.17% | Chr_27 | 19,545,946 | 3.36% |
| Chr_12 | 20,222,205 | 3.48% | Chr_28 | 18,540,835 | 3.19% |
| Chr_13 | 26,128,831 | 4.50% | Chr_29 | 17,464,928 | 3.01% |
| Chr_14 | 22,448,423 | 3.86% | Chr_30 | 20,963,041 | 3.61% |
| Chr_15 | 21,921,918 | 3.77% | Chr_31 | 20,057,418 | 3.45% |
| Chr_16 | 19,815,103 | 3.41% | - | - |  |
| Total chromosome level scaffold length | | | 567,168,877 | | |
| Total length | | | 580,885,107 | | |
| Chromosome/total (%) | | | 97.64% | | |

**Table S6. The statistics of RNA sequencing reads on Illumina platform.** These data are produced by short insert library, and the results were shown by the filtered reads.

| Library | Organ/Tissue | Read number | Total bases | Sequencing strategy |
| --- | --- | --- | --- | --- |
| AN332-01T0001 | Whole body | 34,081,754 | 4,771,445,560 | PE150 |
| Total | - | 34,081,754 | 4,771,445,560 | - |

**Table S7. The statistics of the assembled transcripts by Bridger of 5 organs/tissues.**

| Term | Size (bp) | Number |
| --- | --- | --- |
| N90 | 496 | 35,953 |
| N80 | 900 | 25,192 |
| N70 | 1,307 | 18,529 |
| N60 | 1,718 | 13,692 |
| N50 | 2,155 | 9,929 |
| Max length (bp) | 39,600 | - |
| Total size (bp) | 72,639,419 | - |
| Total number (>1 kb) | - | 23,334 |
| Total number (>10 kb) | - | 94 |

**Table S8. The statistics of the transcripts mapping ratio on the assembled genome.**

| Tissues/Organs | Mapping transcripts number | Total transcripts number | Mapping ratio (%) |
| --- | --- | --- | --- |
| Whole body | 58,742 | 59,695 | 98.40% |

**Table S9.** The quality evaluation of assembled genome by BUSCO software

| Library | Eukaryota | Metazoa | Lepidoptera |
| --- | --- | --- | --- |
| Complete BUSCOs (C) | 246 | 931 | 5,006 |
| Complete and single-copy BUSCOs (S) | 234 | 898 | 4,905 |
| Complete and duplicated BUSCOs (D) | 12 | 33 | 101 |
| Fragmented BUSCOs (F) | 7 | 10 | 136 |
| Missing BUSCOs (M) | 2 | 13 | 144 |
| Total BUSCO groups searched | 255 | 954 | 5,286 |
| Summarize (%) | 96.47% | 97.59% | 94.7% |

**Table S10. Comparison of related species genomes with our chromosome-level genome**.

| Species | Family | Genome source | Assembly level | Genome size | Genome N50 | Scaffold/Chromosome number | BUSCO (eukaryota_odb10) |
| --- | --- | --- | --- | --- | --- | --- | --- |
| *S. cmerearia* | Geometridae | This study | Chromosome | 580,885,107 | 19,566,149 | 105 | 96.5% |
| *O. brumata* | Geometridae | https://www.bioinformatics.nl/wintermoth/portal/data/ | Chromosome | 638,207,720 | 65,630 | 25,801 | 89.0% |
| *H. kahamanoa* | Cosmopterigidae | NCBI (GCF_003589595.1) | Scaffold | 731,440,758 | 13,520,097 | 2,929 | 78.8% |
| *C. medinalis* | Crambidae | http://www.insect-genome.com/Cmed/ | Chromosome | 528,450,646 | 16,091,296 | 3,248 | 92.6% |
| *G. mellonella* | Pyralidae | NCBI (GCF_003640425.2) | Scaffold | 401,914,826 | 13,483,299 | 13,304 | 88.2% |
| *A. transitella* | Pyralidae | NCBI (GCF_001186105.1) | Scaffold | 406,468,287 | 1,586,980 | 7,301 | 81.2% |
| *C. suppressalis* | Pyralidae | http://www.insect-genome.com/chilo/download.php | Chromosome | 827,923,021 | 27,085,414 | 6,400 | 89.9% |
| *S. frugiperda* | Noctuidae | NCBI (GCF_011064685.1) | Chromosome | 486,287,157 | 16,346,893 | 92 | 88.2% |
| *T. ni* | Noctuidae | NCBI (GCF_003590095.1) | Chromosome | 368,211,209 | 14,205,900 | 1,031 | 98.0% |
| *B. mori* | Bombycidae | NCBI (GCF_014905235.1) | Chromosome | 460,349,660 | 16,796,068 | 697 | 99.2% |
| *M. sexta* | Sphingidae | NCBI (GCF_014839805.1) | Chromosome | 470,036,997 | 14,248,853 | 4,057 | 96.9% |
| *A. pernyi* | Saturniidae | https://ngdc.cncb.ac.cn/search/?dbId=gwh&q=Antheraea+pernyi | Chromosome | 727,309,273 | 13,766,174 | 423 | 95.7% |
| *P. bianor* | Papilionidae | GigaDB | Chromosome | 421,524,737 | 13,120,256 | 1,710 | 94.5% |

**Table S11. The statistics of the annotated repeat sequences in our assembled genome.** The type represents that the way or software used in this study.

| Type | Repeat Size | % of genome |
| --- | --- | --- |
| Trf | 24,150,858 | 4.157596% |
| Repeatmasker | 2,944,249 | 0.506856% |
| Proteinmasker | 28,831,757 | 4.963418% |
| *De novo* | 231,946,406 | 39.929825% |
| Total | 252,828,304 | 43.524666% |

**Table 12. The statistics of the annotated repeat sequences in our assembled genome by *de novo* prediction.**

| Type | Repbase TEs | | TE protiens | | *De novo* | | Combined TEs | |
| --- | --- | --- | --- | --- | --- | --- | --- | --- |
|  | Length (bp) | % in genome | Length (bp) | % in genome | Length (bp) | % in genome | Length (bp) | % in genome |
| DNA | 1,226,837 | 0.211201% | 3,350,091 | 0.576722% | 42,837,075 | 7.374449% | 44,270,854 | 7.621275% |
| LINE | 558,240 | 0.096102% | 16,485,563 | 2.838008% | 35,579,401 | 6.125032% | 37,527,922 | 6.460472% |
| SINE | 316 | 0.000054% | - | - | 14,287,006 | 2.459524% | 14,287,170 | 2.459552% |
| LTR | 1,308,094 | 0.225190% | 6,922,240 | 1.191671% | 10,563,837 | 1.818576% | 13,115,517 | 2.257850% |
| Other | 70,686 | 0.012169% | 2,075,880 | 0.357365% | 25,815,226 | 4.444119% | 26,084,064 | 4.490400% |
| UnKnown | 51,235 | 0.008820% | - | - | 114,821,955 | 19.766724% | 114,873,094 | 19.775527% |
| Total | 2,929,635 | 0.504340% | 28,831,757 | 4.963418% | 231,946,406 | 39.929825% | 236,540,697 | 40.720737% |

**Table S13. The functional annotation of the predicted protein-coding genes.**

| Database | Number | Percentage (%) |
| --- | --- | --- |
| InterPro | 12,645 | 59.15% |
| GO | 8,826 | 41.29% |
| KEGG | 10,590 | 49.54% |
| Swissprot | 12,049 | 56.36% |
| TrEMBL | 18,130 | 84.81% |
| Cog | 5,757 | 26.93% |
| Nr (NCBI) | 18,280 | 85.51% |
| Annotated | 18,472 | 86.41% |
| Unanotated | 2,905 | 13.59% |
| Total | 21,377 | - |

**Table S14. GO enrichment of the expanded gene families in *S. cinerearia* analyzed by CAFÉ (v4.1).**

| GO ID | GO Term | GO Class | *P*-value |
| --- | --- | --- | --- |
| GO:0003676 | nucleic acid binding | MF | 3.86E-53 |
| GO:0097159 | organic cyclic compound binding | MF | 8.99E-36 |
| GO:1901363 | heterocyclic compound binding | MF | 8.99E-36 |
| GO:0003677 | DNA binding | MF | 2.19E-14 |
| GO:0042302 | structural constituent of cuticle | MF | 1.18E-08 |
| GO:0005198 | structural molecule activity | MF | 1.87E-08 |
| GO:0016788 | hydrolase activity, acting on ester bonds | MF | 8.64E-08 |
| GO:0005576 | extracellular region | CC | 3.06E-06 |
| GO:0052689 | carboxylic ester hydrolase activity | MF | 1.68E-05 |
| GO:0008612 | peptidyl-lysine modification to hypusine | BP | 0.0001913 |
| GO:0018193 | peptidyl-amino acid modification | BP | 0.0001913 |
| GO:0018205 | peptidyl-lysine modification | BP | 0.0001913 |
| GO:0046516 | hypusine metabolic process | BP | 0.0001913 |
| GO:0043248 | proteasome assembly | BP | 0.0005138 |
| GO:0006575 | cellular modified amino acid metabolic process | BP | 0.0005375 |
| GO:0042398 | cellular modified amino acid biosynthetic process | BP | 0.0005375 |
| GO:1901605 | alpha-amino acid metabolic process | BP | 0.0005375 |
| GO:1901607 | alpha-amino acid biosynthetic process | BP | 0.0005375 |
| GO:0006520 | cellular amino acid metabolic process | BP | 0.0009953 |
| GO:0006082 | organic acid metabolic process | BP | 0.0009953 |
| GO:0019752 | carboxylic acid metabolic process | BP | 0.0009953 |
| GO:0043436 | oxoacid metabolic process | BP | 0.0009953 |
| GO:0008652 | cellular amino acid biosynthetic process | BP | 0.0011751 |
| GO:0016053 | organic acid biosynthetic process | BP | 0.0011751 |
| GO:0046394 | carboxylic acid biosynthetic process | BP | 0.0011751 |
| GO:0034622 | cellular macromolecular complex assembly | BP | 0.0019326 |
| GO:0043623 | cellular protein complex assembly | BP | 0.0019326 |
| GO:0044283 | small molecule biosynthetic process | BP | 0.0022025 |
| GO:0044711 | single-organism biosynthetic process | BP | 0.0022025 |
| GO:0003674 | molecular_function | MF | 0.0025418 |
| GO:0003993 | acid phosphatase activity | MF | 0.0037568 |
| GO:0016791 | phosphatase activity | MF | 0.0037568 |
| GO:0006461 | protein complex assembly | BP | 0.0085515 |
| GO:0065003 | macromolecular complex assembly | BP | 0.0085515 |
| GO:0070271 | protein complex biogenesis | BP | 0.0085515 |
| GO:1901566 | organonitrogen compound biosynthetic process | BP | 0.0085554 |
| GO:0044281 | small molecule metabolic process | BP | 0.0089246 |
| GO:0042578 | phosphoric ester hydrolase activity | MF | 0.0122111 |
| GO:0043933 | macromolecular complex subunit organization | BP | 0.0140838 |
| GO:0071822 | protein complex subunit organization | BP | 0.0140838 |
| GO:1901564 | organonitrogen compound metabolic process | BP | 0.0183311 |

**Table S15. KEGG enrichment of the expanded gene families in *S. cinerearia* analyzed by CAFÉ (v4.1).**

| Map ID | Pathway | Count | *P*-value |
| --- | --- | --- | --- |
| map00360 | Phenylalanine metabolism | 19 | 4.76E-05 |
| map00073 | Cutin, suberine and wax biosynthesis | 11 | 0.000441887 |
| map04614 | Renin-angiotensin system | 10 | 0.000734574 |
| map01200 | Carbon metabolism | 36 | 0.000991797 |
| map05204 | Chemical carcinogenesis | 28 | 0.001092151 |
| map04962 | Vasopressin-regulated water reabsorption | 13 | 0.001624295 |
| map05321 | Inflammatory bowel disease (IBD) | 4 | 0.005030537 |
| map02060 | Phosphotransferase system (PTS) | 8 | 0.006181982 |
| map04145 | Phagosome | 21 | 0.006694331 |
| map05132 | Salmonella infection | 14 | 0.006783499 |
| map04622 | RIG-I-like receptor signaling pathway | 8 | 0.007482992 |
| map01100 | Metabolic pathways | 200 | 0.008878046 |
| map01212 | Fatty acid metabolism | 22 | 0.00945799 |
| map04610 | Complement and coagulation cascades | 8 | 0.012628194 |
| map04810 | Regulation of actin cytoskeleton | 27 | 0.017850989 |
| map04916 | Melanogenesis | 14 | 0.027158794 |
| map01110 | Biosynthesis of secondary metabolites | 76 | 0.039221707 |
| map00350 | Tyrosine metabolism | 13 | 0.046976715 |
| map04914 | Progesterone-mediated oocyte maturation | 17 | 0.048264741 |
| map05130 | Pathogenic Escherichia coli infection | 10 | 0.04855159 |

**Table S16. Relative evolution rate among these species by LINTRE software.**

| Outgroup | Ingroup1 | Ingroup2 | bA | bB | delta | Z score | CP (%) | Faster |
| --- | --- | --- | --- | --- | --- | --- | --- | --- |
| *P. bianor* | *S. cmerearia* | *S. frugiperda* | 0.218192 | 0.129096 | 0.089096 | 73.789970 | 99.96 | *S. cmerearia* |
|  |  | *A. pernyi* | 0.215430 | 0.158992 | 0.056437 | 43.665972 | 99.96 | *S. cmerearia* |
|  |  | *M. sexta* | 0.218300 | 0.126944 | 0.091356 | 76.107398 | 99.96 | *S. cmerearia* |
|  |  | *A. transitella* | 0.225193 | 0.150003 | 0.075190 | 60.129186 | 99.96 | *S. cmerearia* |
|  |  | *G. mellonella* | 0.226126 | 0.136654 | 0.089472 | 71.656373 | 99.96 | *S. cmerearia* |
|  |  | *B. mori* | 0.219277 | 0.159515 | 0.059763 | 47.578113 | 99.96 | *S. cmerearia* |
|  |  | *T. ni* | 0.217080 | 0.123436 | 0.093644 | 78.314600 | 99.96 | *S. cmerearia* |
|  |  | *H. kahamanoa* | 0.226446 | 0.155246 | 0.071200 | 56.326153 | 99.96 | *S. cmerearia* |
|  |  | *C. medinalis* | 0.222620 | 0.141394 | 0.081226 | 64.388768 | 99.96 | *S. cmerearia* |
|  |  | *O. brumata* | 0.181102 | 0.160890 | 0.020212 | 15.175083 | 99.96 | *S. cmerearia* |

**Table S17. Relative evolution rate among these species by MEGA software.**

| Outgroup | Ingroup1 | Ingroup2 | Identical | Ingroup1  specific | Ingroup2  specific | Chi-score | P-value | Faster |
| --- | --- | --- | --- | --- | --- | --- | --- | --- |
| *P. bianor* | *S. cmerearia* | *S. frugiperda* | 270,968 | 42,233 | 23,112 | 5595.11 | <0.000001 | *S. cmerearia* |
|  |  | *A. pernyi* | 248,903 | 37,901 | 26,742 | 1926.32 | <0.000001 | *S. cmerearia* |
|  |  | *M. sexta* | 272,058 | 42,471 | 22,752 | 5961.68 | <0.000001 | *S. cmerearia* |
|  |  | *A. transitella* | 266,389 | 42,504 | 26,558 | 3681.84 | <0.000001 | *S. cmerearia* |
|  |  | *G. mellonella* | 261,232 | 42,361 | 23,699 | 5272.03 | <0.000001 | *S. cmerearia* |
|  |  | *B. mori* | 266,854 | 41,133 | 28,506 | 2289.54 | <0.000001 | *S. cmerearia* |
|  |  | *T. ni* | 272,417 | 42,476 | 22,246 | 6323.24 | <0.000001 | *S. cmerearia* |
|  |  | *H. kahamanoa* | 264,555 | 42,572 | 27,538 | 3223.81 | <0.000001 | *S. cmerearia* |
|  |  | *C. medinalis* | 257,788 | 41,352 | 24,633 | 4236.19 | <0.000001 | *S. cmerearia* |
|  |  | *O. brumata* | 227,596 | 29,180 | 25,625 | 230.60 | <0.000001 | *S. cmerearia* |

1. Tajima F. (**1993**). Simple methods for testing molecular clock hypothesis. *Genetics***135**:599-607.

2. Kumar S., Stecher G., Li M., Knyaz C., and Tamura K. (**2018**). MEGA X: Molecular Evolutionary Genetics Analysis across computing platforms. *Molecular Biology and Evolution* **35**:1547-1549.

3. Stecher G., Tamura K., and Kumar S. (**2020**). Molecular Evolutionary Genetics Analysis (MEGA) for macOS. *Molecular Biology and Evolution* (https://doi.org/10.1093/molbev/msz312).

**Table S18. Statistics of positively selected genes in Geometridae.**

| Gene name | A_w | B_w | S_w | delta_lnl | *P*-value | Adjusted *P-value* |
| --- | --- | --- | --- | --- | --- | --- |
| *GOLGA2* | 0.07058 | 0.06684 | 999 | 12.187364 | 0.000481143 | 0.014484937 |
| *Dlish* | 0.00460 | 0.00393 | 137.62177 | 14.089616 | 0.000174303 | 0.007385283 |
| *Rab35* | 0.00558 | 0.00540 | 1.86071 | 11.151586 | 0.000839599 | 0.020790145 |
| *Smg5* | 0.07881 | 0.07660 | 999 | 23.191850 | 0.000001466 | 0.000712426 |
| *Zeb2* | 0.08975 | 0.08907 | 999 | 12.432896 | 0.000421837 | 0.014193574 |
| *Tango1* | 0.07676 | 0.07303 | 999 | 17.355198 | 0.000031005 | 0.002817760 |
| *CHORD* | 0.05393 | 0.05065 | 2.57553 | 12.853470 | 0.000336854 | 0.012430999 |
| *SAK* | 0.04506 | 0.04399 | 999 | 9.155858 | 0.002479231 | 0.045019687 |
| *ttc37* | 0.04705 | 0.04549 | 999 | 8.992628 | 0.002710709 | 0.047708478 |
| *MROH1* | 0.04798 | 0.04632 | 999 | 22.173482 | 0.000002491 | 0.000712426 |
| *Kat8* | 0.01328 | 0.01237 | 205.46795 | 15.609568 | 0.00007786 | 0.004687992 |
| *GET4* | 0.04484 | 0.04284 | 999 | 10.441272 | 0.001232304 | 0.027110688 |
| *Aaas* | 0.05862 | 0.05537 | 999 | 14.745256 | 0.000123057 | 0.005865717 |
| *Sox15* | 0.02355 | 0.02161 | 999 | 13.289338 | 0.00026692 | 0.010529534 |
| *PNLIPRP2* | 0.07686 | 0.07338 | 999 | 9.379600 | 0.002194134 | 0.041149005 |
| *FAM120A* | 0.05277 | 0.05054 | 999 | 13.571006 | 0.000229706 | 0.009385131 |
| *Dchs1* | 0.08235 | 0.08129 | 999 | 9.455092 | 0.002105637 | 0.040147479 |
| *ANKRD13C* | 0.02888 | 0.02756 | 999 | 15.232922 | 0.000095032 | 0.005176981 |
| *MED14* | 0.06422 | 0.06235 | 3.02606 | 11.799270 | 0.00059254 | 0.016946644 |
| *SUCLA2* | 0.05626 | 0.05184 | 810.04379 | 21.427754 | 0.000003674 | 0.000806520 |
| *NISCH* | 0.04725 | 0.04497 | 999 | 12.075700 | 0.000510833 | 0.014984435 |
| *TM-A2B* | 0.04674 | 0.04480 | 999 | 11.630646 | 0.00064874 | 0.018101428 |
| *ARFIP1* | 0.03078 | 0.02775 | 999 | 10.147590 | 0.0014449 | 0.031170716 |
| *tipE* | 0.03268 | 0.03002 | 999 | 12.335114 | 0.000444517 | 0.014484937 |
| *gk5* | 0.06540 | 0.06328 | 999 | 12.212014 | 0.000474827 | 0.014484937 |
| *march5* | 0.02437 | 0.02203 | 999 | 12.251318 | 0.00046493 | 0.014484937 |
| *btbd6* | 0.03053 | 0.02878 | 999 | 20.606664 | 0.00000564 | 0.000806520 |
| *UBFD1* | 0.05619 | 0.05336 | 999 | 8.891850 | 0.002864464 | 0.049650709 |
| *PHF3* | 0.05439 | 0.05357 | 999 | 15.468278 | 0.000083902 | 0.004799194 |
| *Uty* | 0.03719 | 0.03439 | 4.32998 | 22.835196 | 0.000001765 | 0.000712426 |
| *Tl* | 0.12241 | 0.11794 | 999 | 10.044368 | 0.001528144 | 0.031785395 |
| *ChT* | 0.03133 | 0.02982 | 999 | 18.767324 | 0.000014768 | 0.001535872 |
| *LARS* | 0.02919 | 0.02814 | 999 | 11.228336 | 0.000805578 | 0.020790145 |
| *Dis3l2* | 0.05801 | 0.05562 | 999 | 14.558946 | 0.000135842 | 0.005977048 |


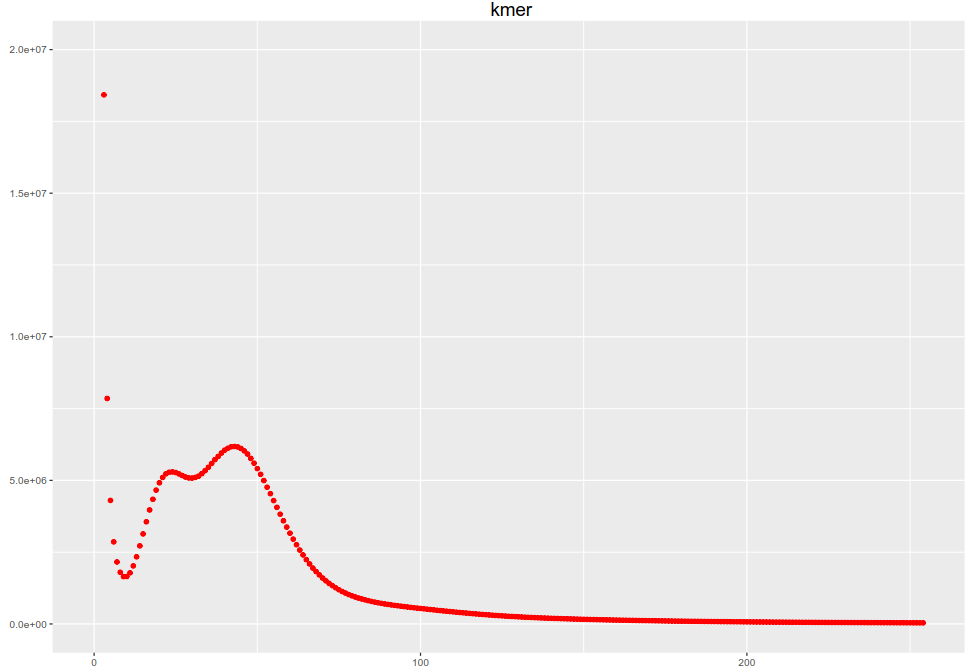


**Figure S1. 17-mer analysis of *S. cinerearia* genome.**


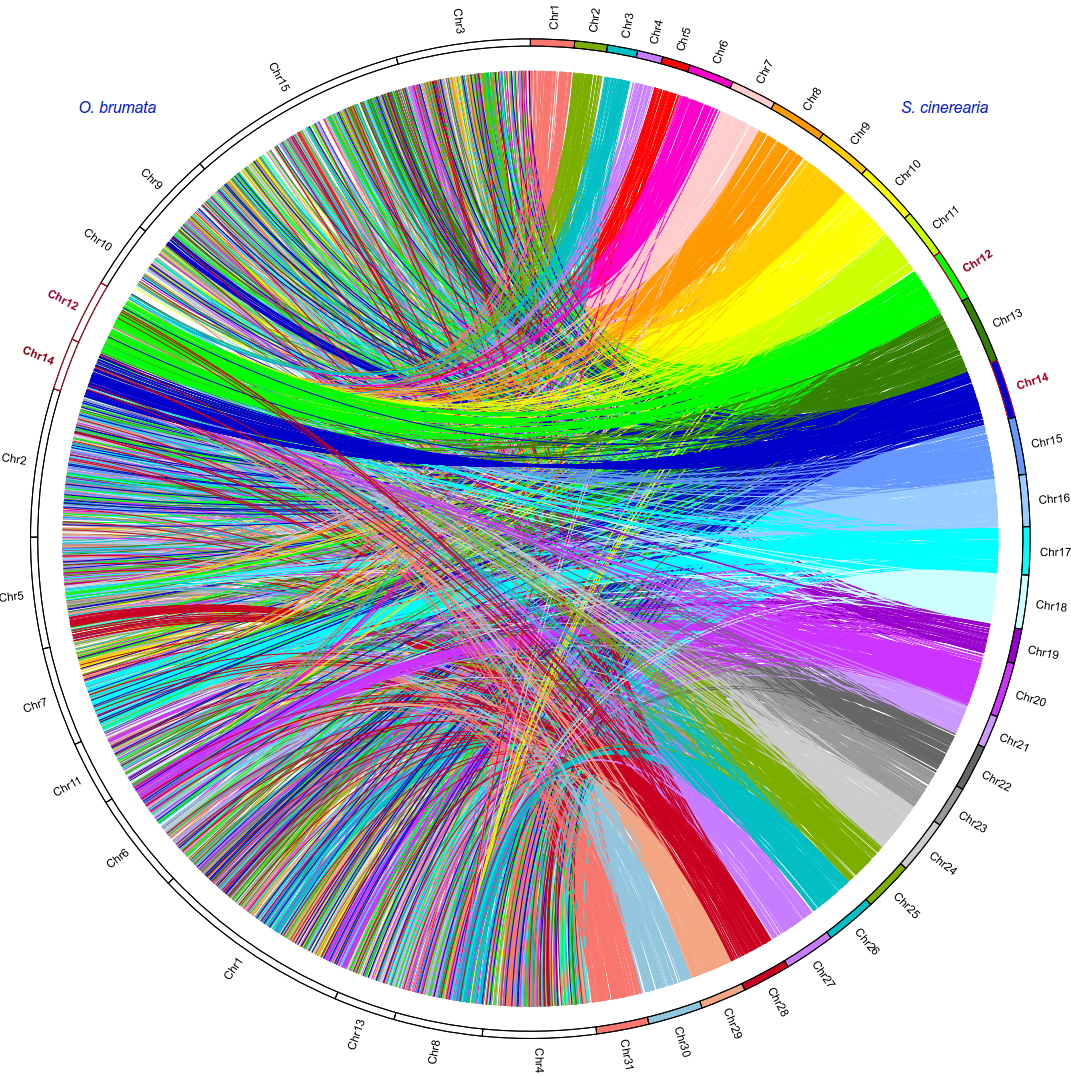


**Figure S2.** Whole genome synteny analyses between *S. cinerearia* and *O. brumata*.


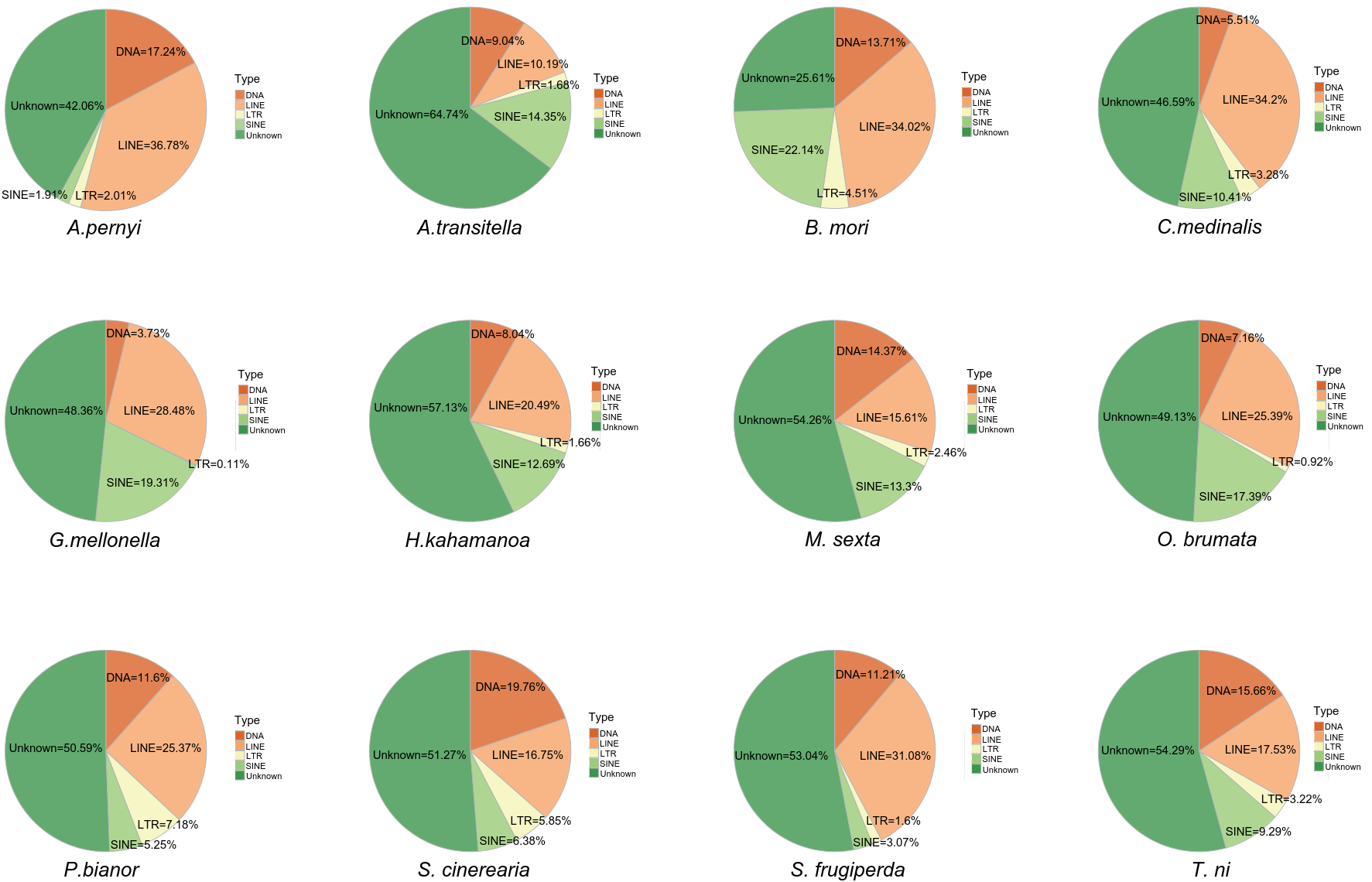


**Figure S3. TEs ratio in these species.**


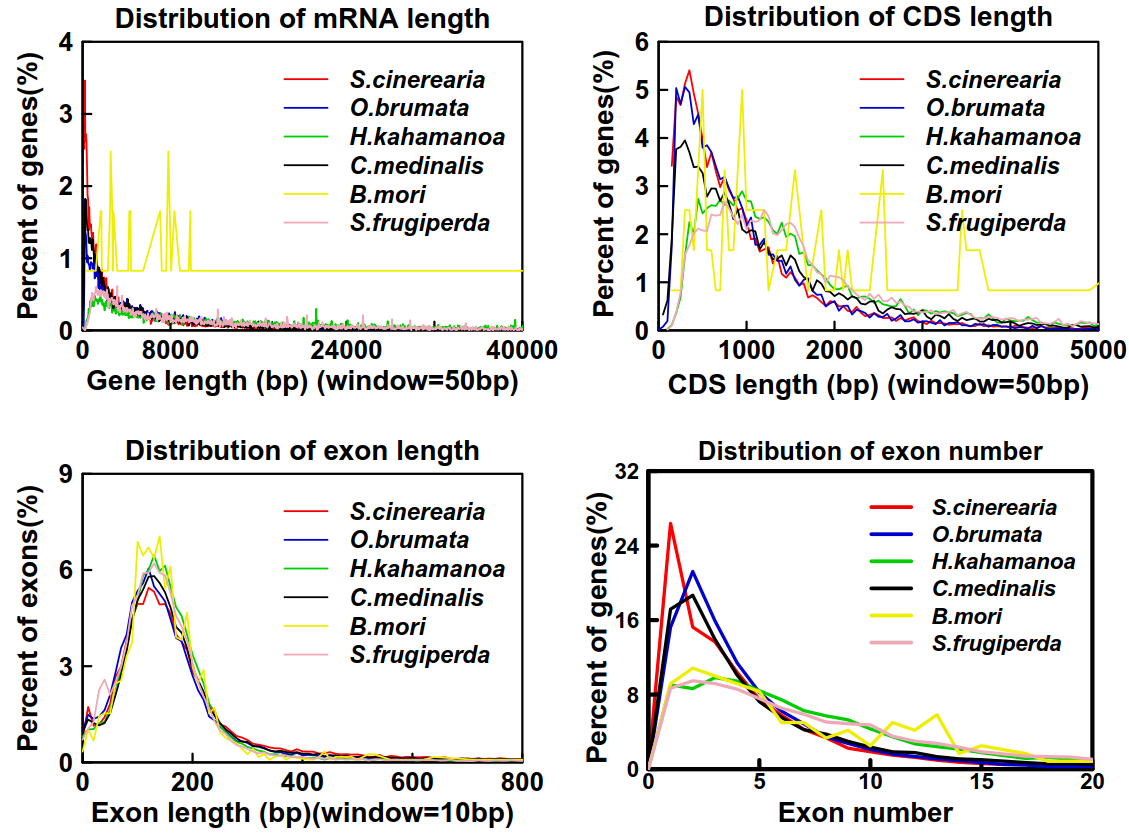


**Figure S4. Distribution of gene parameters in these species.**


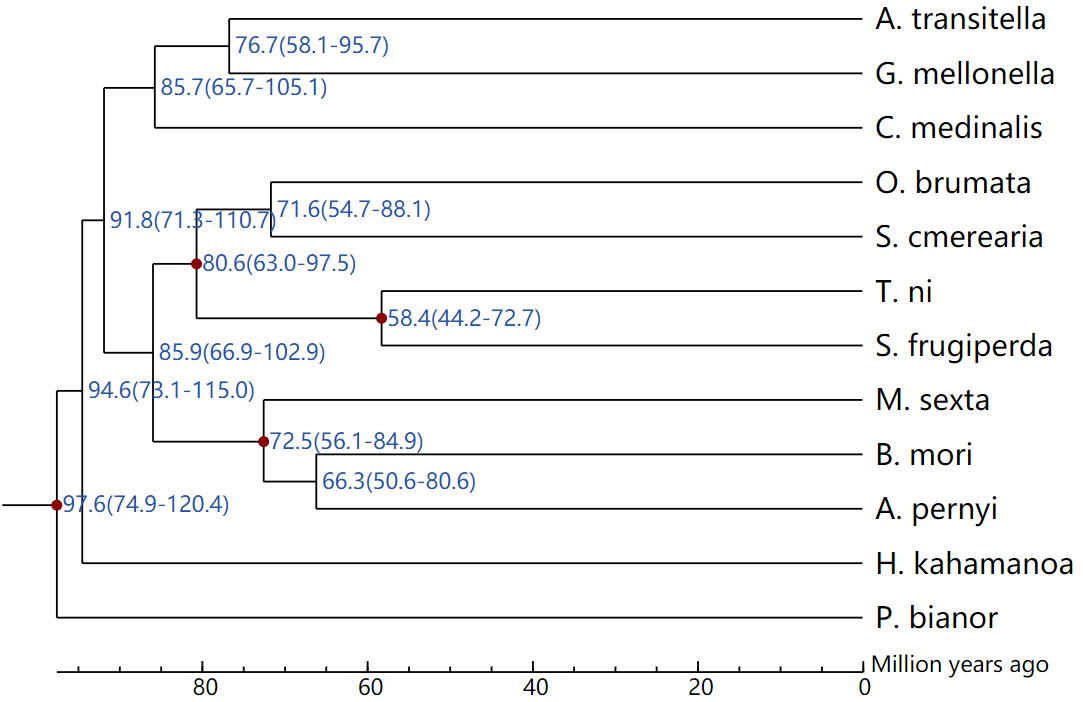


**Figure S5. Divergence time of these species.**


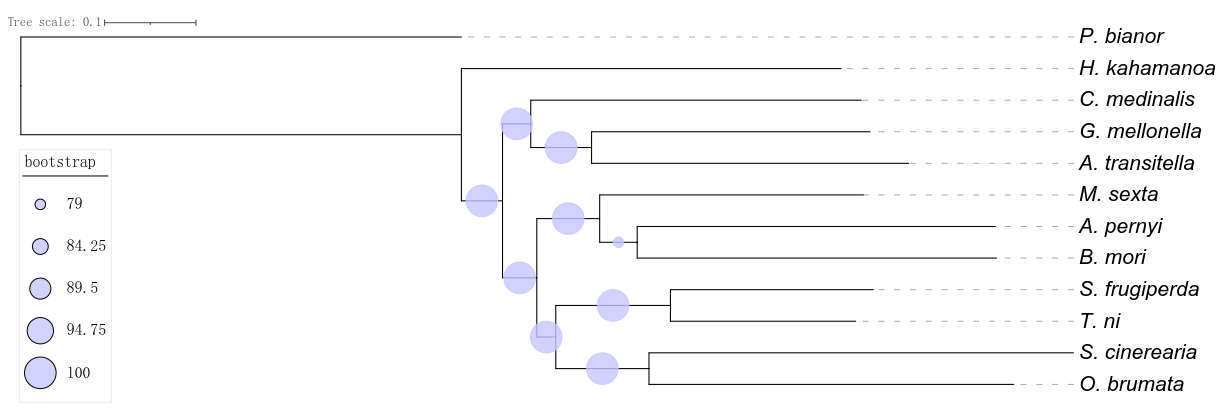


**Figure S6. Phylogenetic relationship among the 12 species inferred by the nucleotide acid sequences of the single-copy genes.** Number in the node represents the corresponding bootstrap value.
